# Can Orthodontic Adhesive Systems Inhibit the Formation and Development of White Spot Lesions During Fixed Orthodontic Treatment? A Systematic Review

**DOI:** 10.3290/j.jad.b5781299

**Published:** 2024-10-14

**Authors:** Marwan El Helou, Sandra Chakar, Emmanuel Nicolas, Elias Estephan, Frederic Cuisinier, Stéphane Barthélemi

**Affiliations:** a Senior Lecturer and Hospital Practitioner, Faculty of Dentistry, Centre for Research in Clinical Dentistry (CROC), University of Clermont Auvergne BP 10448, Clermont-Ferrand, France; CHU Clermont-Ferrand, Service d’Odontologie, CHU Estaing, Clermont-Ferrand, France. Conducted research, collected data, and wrote the article.; b Dentist, private practice, Abu Dhabi, United Arab Emirates. Participated in research, data extraction, and writing.; c Dean, Faculty of Dentistry, Centre for Research in Clinical Dentistry (CROC), University of Clermont Auvergne BP 10448, Clermont-Ferrand, France; CHU Clermont-Ferrand, Service d’Odontologie, CHU Estaing, Clermont-Ferrand, France. Reviewed and corrected the manuscript.; d Associate Professor, Faculty of Dentistry, University of Montpellier, France; Head of the Bioengineering and Nanoscience Laboratory, University of Montpellier; France. Critically reviewed the article.; e Professor and Hospital Practitioner, Faculty of Dentistry, Montpellier; Head of the Bioengineering and Nanoscience Laboratory, Dental School, University of Montpellier, France. Provided feedback and assisted with methodology.; f Professor and Hospital Practitioner; Head of the Orthodontics Department, Faculty of Dentistry, Dental School, University of Montpellier, France. Coordinated the efforts of all authors.

**Keywords:** enamel demineralization, fixed orthodontics, orthodontic adhesive, prevention, white spot lesion

## Abstract

**Purpose:**

This study aims to assess whether orthodontic bonding systems prevent orthodontic-induced white spot lesions (OIWSLs), exploring efficacy and identifying associated factors through a comprehensive systematic review of existing evidence.

**Materials and Methods:**

The study complied to Preferred Reporting Items for Systematic Reviews and Meta-Analyses (PRISMA) guidelines. Two evaluators screened records, and data were extracted on orthodontic bonding systems, outcomes, and participant characteristics from PubMed/MEDLINE, Cochrane Library, and EM Premium. The search equation focused on white spot lesions and orthodontic bonding. Only *in-vivo* studies and clinical trials on humans were included, while *in-vitro* studies were excluded. The risk of bias was assessed using Cochrane’s RoB2 tool for RCTs and ROBINS-I tool for non-randomized studies, evaluating key domains related to bias.

**Results:**

The systematic review, including 12 articles with 550 participants and 2,000 teeth, revealed that bonding with nanoparticles of nCaF_2_-primer and amorphous calcium phosphate-containing adhesives effectively reduced WSLs. In contrast, one-step adhesive without primer (GC Ortho Connect^™^) was associated with higher and more severe WSLs. Fluoride-releasing primers (Opal Seal^™^ and Clearfil^™^) did not exhibit an advantage in demineralization reduction. The inclusion of TiO_2_ nanoparticles in two studies yielded conflicting results on antibacterial effects.

**Discussion:**

Various nanoparticles incorporated into adhesives or primers exhibit promise in preventing white spot lesions in fixed orthodontic treatment. However, the used evaluation methods, such as clinical examinations or advanced imaging, significantly impact result interpretation. The effectiveness of orthodontic adhesives in preventing WSLs should balance between biocompatibility, bond strength and demineralization control tailored to patient-specific needs.

Orthodontic induced white spot lesions (OIWSLs), indicative of enamel demineralization, have long been a prevalent concern during fixed orthodontic treatment. These lesions, which manifest as milky white opaque areas on teeth, pose esthetic and oral health challenges during and after treatment.^22^ OIWSLs are associated with a wide range of orthodontic patients, with prevalence estimates spanning from 11% to as high as 46%.^38^ The occurrence of white spot lesions is associated with various factors, including plaque accumulation, dietary habits, salivary composition, and changes in the oral bacterial environment, particularly during orthodontic treatment. As plaque bacteria generate acidic byproducts, enamel demineralization arises, leading to the formation of WSLs. These lesions typically appear within 4 weeks of treatment initiation and can affect a broad spectrum of orthodontic patients, predominantly occurring on the labio-gingival surfaces of lateral incisors, with males displaying a higher susceptibility.^33^ Orthodontic appliances including brackets, wires, and bands create retention sites for plaque, significantly elevating the risk of WSL formation, particularly in adolescents undergoing fixed orthodontics. Additionally, adhesives used for bracket bonding can create irregularities or gaps where plaque and bacteria can accumulate more readily.^21^

While various treatment methods after appliance removal have been proposed, including microabrasion and resin infiltration,^28^ the most effective approach still lies in the prevention of their occurrence through good oral hygiene practices and the application of remineralizing agents such as fluoride and casein phosphopeptide-amorphous calcium phosphate (CPP-ACP).^1^ However, the effectiveness of in-office topical applications or home rinse programs is hindered by drawbacks such as extended chair time at the office or unpredictable patient compliance.^9^

*In-vitro* studies have shown that incorporating antimicrobial agents in the adhesives helps inhibit microbial growth, particularly of cariogenic bacteria like *Streptococcus mutans*. Additionally, biopolymers exhibit strong antimicrobial properties and may further reduce plaque formation and the risk of enamel demineralization, thereby possibly inhibiting the development of white spot lesions during orthodontic treatment.^42^

Despite persistent efforts to prevent the clinical occurrence of WSLs, it remains uncertain whether the bonding methods or bonding agents included in the composite resin or primer used in fixed orthodontics can halt the appearance and progression of OIWSLs. Although this issue has a profound clinical significance, a comprehensive systematic review that explores the relationship between orthodontic bonding systems and WSL formation and development is absent.

The objective of this systematic review is to synthesize findings from clinical trials in order to answer the following questions: Can orthodontic bonding methods or bonding additives effectively prevent the emergence and progression of WSLs *in vivo*, and if so, what specific characteristics or factors contribute to their efficacy? By rigorously analyzing the available evidence, our aim is to provide answers about a potential connection between orthodontic bonding systems and WSLs.

## Materials and Methods

### Protocol and Registration

This systematic review adhered to the 2020 statement of the Preferred Reporting Items for Systematic Reviews and Meta-Analyses (PRISMA) guidelines.^26^ Prior to commencing the review process, the authors formulated a comprehensive methodology protocol. Furthermore, to ensure transparency and credibility, this review was registered on the CRD York website PROSPERO and assigned the protocol number CRD42023460183.

### Eligibility Criteria

#### Inclusion criteria

1. *Study design:* only interventional studies on humans will be included (*in-vivo* studies, controlled trials and RCTs).
2. *Participants:* studies involving young patients undergoing fixed orthodontic treatment, regardless of gender will be included.
3. *Intervention:* studies investigating the effects of different orthodontic bonding systems (adhesive materials, bonding protocols, etc.) on the formation and development of white spot lesions will be included.
4. *Comparison:* studies must include at least two different types of orthodontic bonding systems for comparison purposes. This could involve variations in adhesive materials, bonding protocols, or other bonding-related factors.
5. *Outcome:* the primary outcome of interest is the occurrence, severity, or progression of white spot lesions. Studies reporting objective measures, such as clinical examinations, radiographic assessments, or quantitative assessments of lesion size and depth, will be included.
6. *Language:* studies published in English or French will be considered to ensure effective comprehension and interpretation.
7. *Date:* Only studies published within the last 5 years will be included.

#### Exclusion criteria

Non-interventional study designs such as observational studies, case reports, and non-clinical research were to be excluded. The review would also exclude animal studies as well as topics unrelated to fixed orthodontic treatment, including the formation of WSLs in removable aligners, lingual brackets, or around fixed retainers. Furthermore, studies investigating the effects of topical varnish application around braces would not be considered. To prevent data duplication, only the most comprehensive or recent publication of the same randomized controlled trial would be incorporated.

#### Information sources

The search to identify relevant studies was conducted using the following sources, with searches extending up to October 2023 for each: PubMed/MEDLINE, Cochrane Library (including CENTRAL), and EM Premium.

### Search Strategy

The search strategy involved a combination of relevant keywords and MeSH terms related to orthodontic bonding systems and white spot lesions. The search equation was adapted to match the specific syntax and indexing of each database. For PubMed/MEDLINE, the equation was (white spot lesion OR dental white spot) AND (orthodontic bonding OR orthodontic adhesive OR fixed orthodontic). In Cochrane Library (including CENTRAL), the equation was ((“white spot lesion” OR “dental white spot”) AND (“orthodontic bonding” OR “orthodontic adhesive” OR “fixed orthodontic”)). Similarly, in EM Premium, the equation used was ((white spot lesion OR dental white spot) AND (orthodontic bonding OR orthodontic adhesive OR fixed orthodontic)). The last search was conducted in October 2023, and all studies identified up to that date were considered for inclusion in the review.

### Selection and Data Collection Process

To determine whether a study met the inclusion criteria for the review, a two-step screening process was employed by two independent reviewers (MEH and SC). Initially, a preliminary triage was conducted based on the title and abstract of each record. If a paper appeared to address the research question during this initial assessment, a full reading of the paper was performed to make the final decision regarding its inclusion or exclusion in the review. Data was subsequently collected by the same two independent reviewers. Any disagreements between the two reviewers regarding the inclusion or exclusion of specific information or articles were resolved through discussion and consensus.

### Data Extraction and Results Presentation

The outcomes for which data were sought were as follows:

#### Primary outcome:

Orthodontic bonding system efficacy in preventing white spot lesions.

#### Secondary outcomes:

White spot lesion occurrence and prevalence in patients undergoing orthodontic treatment.

Participant characteristics: data on participant demographics, including age, gender, and baseline oral health conditions.

Intervention characteristics: details of the orthodontic bonding systems used, including the type, treatment duration, and any specific protocols followed.

Data collection was based on the information present in the included studies, and if any outcome data were not reported, this was noted as a limitation in the article.

The data extraction process from the selected studies involved the collection and tabulation of the following information: study titles, author(s), publication year, study design, intervention, evaluation method, results, and authors’ conclusions.

### Risk of Bias Assessment

To assess the study quality according to research designs, specific tools were employed for each study type. For randomized clinical trials, we utilized Cochrane’s revised tool for assessing the risk of bias (RoB), which assesses key domains such as randomization, allocation concealment, blinding, and others to gauge the risk of bias.^35^ In the case of non-randomized studies of interventions, we employed the ROBINS-I tool, which comprehensively evaluates study quality across domains like bias in selection of participants, bias in measurement of outcomes, and bias in the selection of reported results.^34^

## Results

### Study Selection

**Fig 1:**
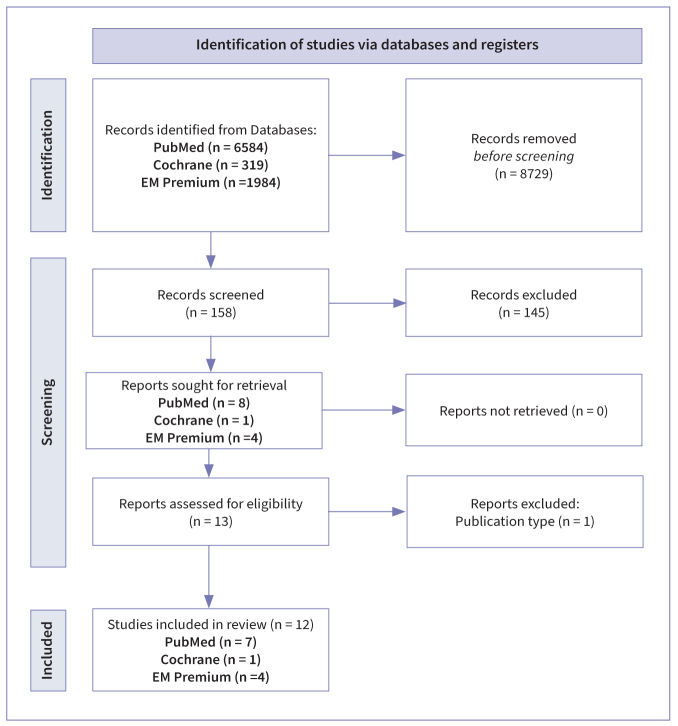
Flowchart

Using our defined search equation, the search process began by identifying 158 records through the examination of titles. Subsequently, abstracts were reviewed for relevance, leading to the selection of 13 records for full-text assessment. During the full-text assessment phase, one study was excluded as it was in fact an *in-vitro* study, which did not align with our predefined inclusion criteria.^18^ Ultimately, 12 studies were included in our systematic review.^12–25^ The flowchart is shown in Figure 1.

### Study Characteristics

Table 1 gives a concise overview of the outcomes and specific findings for each study.

**Table 1:** Summary of study characteristics and outcomes

Author, year of publication, reference number	Study design	Objective(s)	Participants	Intervention	Evaluation methods	Outcome	Authors’ conclusions
Al Tuma et al 2023^3^	Single-center, double-blinded, split-mouth, randomized clinical trial	To compare enamel demineralization around orthodontic brackets bonded with nCaF_2_ primer versus a control primer	31 patients aged 17.9 ± 2.45 years (7 males, 24 females) for a total of 310 teeth evaluated	Dental arches were divided into four quadrants, with random allocation of either the nCaF_2_ primer or the control Transbond primer to each quadrant	DIAGNOdent pen scores (before treatment, at 1, 3, and 6 months). Streptococcus mutans count (at 1, 3, and 6 months) Photographic images (before and after treatment)	Statistically significant scores in favor of the nCaF_2_ primer at T1, T3, and T6 (p-value = 0.000). Statistically significant difference in bacterial count between the two primers only at T1 (p-value = 0.000) No significant differences in the WSLs photographic scores of the control group with the contralateral nCaF_2_ group before and after bonding	nCaF_2_ primer reduces demineralization and *S. mutans* count but no significant difference at a clinical level after bracket debonding
Alshammari et al 2019^6^	Triple-blinded randomized clinical trial	To assess the clinical impact of an adhesive containing amorphous calcium phosphate (ACP) on the reduction of WSL around orthodontic brackets over a 6-month duration	26 patients aged 12–35 years (12 males and 14 females) for a total of 255 teeth evaluated	The dental arches were divided into four quadrants, and each of the six anterior teeth in these quadrants was randomly assigned either the ACP light-cure adhesive or the control Transbond XT adhesive	DIAGNOdent pen scores were obtained immediately after bonding (T0), at 1-month post-bonding (T1), and at 6 months post-bonding (T2), at mesial, distal, incisal, and gingival sites For each group, the demineralization variation (ΔD) was calculated as the change from the baseline demineralization score (T0) to the highest score observed at either T1 or T2	At T1, the control group had an average demineralization score of 3.39 (±1.29), whereas the ACP group had a notably lower average score of 2.58 (±1.11) (p-value of 0.000) At T2, the control group had an average score of 4.32 (±2.32), while the ACP group continued to exhibit a lower average score of 3.41 (±1.11) (p-value of 0.000)	ACP-containing adhesives reduce enamel demineralization in orthodontic treatment compared to regular adhesives
Atilla et al 2020^7^	Single-center, single-blind, two-arm parallel randomized controlled trial	To quantitatively assess the impact of indirect bracket bonding utilizing a flowable composite material, on the formation of WSL	51 patients aged 14.73 ± 1.71 years (26 males and 25 females)	Patients were randomly divided into two groups: the control group received direct bonding (DB) using the Transbond XT adhesive, while the intervention group received indirect bonding (IB) using a flowable adhesive, Opal Bond MV	A quantitative light-induced fluorescence (QLF) camera system was used before and after treatment (T0 and T1). Image analysis with specialized software assessed four parameters: - ΔF (%): Percentage of fluorescence loss indicating lesion depth. - ΔFmax (%): Loss of maximum fluorescence intensity - ΔQ (%px²): Product of lesion area representing lesion volume - WS Area (px²): Lesion area with ΔF ≤ 5%	Increased demineralization in DB Group: The DB group exhibited significantly higher loss of fluorescence and degree of demineralization compared to the IB group between T0 and T1, with notable differences in specific quadrants (p <0.05 for ΔF and ΔF max) Lesion size and volume differences: The DB group displayed a significant loss of fluorescence in maxillary lateral incisors and larger lesion areas (WS area) than the IB group. Additionally, the mandibular left quadrant showed increased loss of fluorescence and degree of demineralization in the DB group (p < 0.05 for ΔQ and WS area)	Indirect bonding and flowable composites reduce WSL formation more effectively
Benson et al 2019^8^	Multicenter, single-blind, randomized controlled trial	To evaluate the impact of resin-modified glass-ionomer cement (RM-GIC) on reducing WSLs during fixed orthodontic treatment. To compare bracket failure rates between RM-GIC and composite resin	197 patients aged 15.5 ± 3.3 years	Patients were randomly divided into two groups: the control group where brackets were bonded with resin adhesive (Transbond XT), while the intervention group was bonded using light-cured RM-GIC	Three sets of digital photographic images taken before and after treatment (right buccal segment, left buccal segment, and frontal view) First-time bond failure counts anterior to first molars	No differences were observed in the proportions of patients experiencing new WSLs or first-time bracket failures when participants were bonded with either resin adhesive or RM-GIC (p > 0.05)	Resin-modified glass ionomer doesn’t reduce new WSLs or cause less bond failures
Comert et al 2020^12^	Prospective clinical study	To compare the effectiveness of a fluoride-filled primer (Opal Seal) and a conventional primer in preventing WSLs. To evaluate the survival rate of orthodontic brackets bonded with both primers	56 patients divided into two groups. Group 1 consisted of 28 patients, with an average age of 15.8 years. Group 2 included 28 patients, with an average age of 14.9 years. In total, 560 tooth surfaces were studied in each group	Group 1 received Opal Seal primer during orthodontic bonding Group 2 received a conventional primer (Transbond XT primer) The same adhesive and brackets were applied in both groups	WSL severity assessment: Digital images were used, and WSL severity was rated from 0 to 3 WSL Severity Score: The DIAGNOdent device provided numerical scores for assessing WSL severity WSL area calculation: Imaging software calculated the areas of WSLs Clinical failure rate: Records were kept for first-time bracket failures during treatment	WSL severity in digital photographs: no significant difference between the two groups (p >0.05) DIAGNOdent measurements: Opal Seal group showed fewer WSLs compared to the Transbond group, with initial demineralization rates of 1.3% and 3.9%, and caries lesion rates of 0.2% and 1.3%, respectively (p <0.03) WSL Area Calculation: There was no significant difference in the WSL area between the two groups, with the mean WSL area measuring 161.3 mm^2^ in the Opal Seal group and 1361.2 mm^2^ in the conventional group Bracket failure rates: No significant differences were observed in bracket failure rates among different primers, dental arches, bracket types, or sexes	Fluoride-releasing primer shows no advantage in reducing demineralization over control
Farzanegan et al 2021^14^	Double-blind randomized clinical trial	To assess the impact of incorporating chitosan nanoparticles (NPs) and TiO_2_ NPs into orthodontic adhesive on Streptococcus mutans counts and enamel mineral content during fixed orthodontic treatment	24 patients were equally divided into two groups. The article does not specify age or gender details of participants	Control group: Bracket bonding using Transbond XT adhesive. Experimental group: bracket bonding was conducted with Transbond XT containing 1% chitosan and 1% TiO_2_ NPs. Specifically, the study focused on a total of 48 upper second premolars and 48 maxillary lateral incisors	*S. mutans* count measurement: Real-time PCR on enamel samples from premolars and incisors Enamel mineral content measurement: Utilization of VistaCam iX camera for quantifying enamel fluorescence. Assessment of enamel health at 8 points near brackets. Both evaluations were made at 1 day, 2 months, and 6 months after bonding	*S. mutans* count measurement: Control group: no significant difference between all timepoints for both areas Experimental group: significant decrease in *S. mutans* count was observed at the time points of 1 day, 2 months, and 6 months for both maxillary lateral incisor (p = 0.01) and maxillary second premolar teeth (p = 0.001) Enamel mineral content: there were no significant differences in enamel mineral content between the experimental group and control group at all time points	Chitosan and TiO_2_ nanoparticles in orthodontic composites exhibit antibacterial properties
Mollabashi et al 2022^23^	Split-mouth randomized controlled clinical trial	To evaluate the impact of a TiO_2_ nanoparticle-modified composite on reducing Streptococcus mutans population and preventing WSLs around brackets	40 patients aged 12–25 years receiving fixed orthodontic treatment	This intervention involved bonding TiO_2_-modified composite (1 wt.%) to lateral incisor brackets and evaluating its effects on reducing *S. mutans* and cytotoxicity over a 6-month period in comparison to a control quadrant	*S. mutans* colony count (PCR) DIAGNOdent score These evaluations were conducted at four time points (T0, T1, T3, and T6) over a period of one to 6 months after bonding orthodontic brackets	*S. mutans* colony count (PCR) No statistically significant difference between the *S. mutans* populations on the control and test sides at any time point DIAGNOdent score Mean readings were significantly greater on the control side than on the experimental side (p < 0.001) at all time points except at baseline	TiO_2_ nanoparticles in orthodontic composites may prevent bracket-induced demineralization; antibacterial effects insignificant
Horan et al 2023^17^	Single-center, double-blind, three-arm, randomized clinical trial	To compare the development of WSLs during fixed orthodontic therapy among a conventional three-step bonding system, a self-etching primer bonding system, and a one-step adhesive bonding system	75 healthy patients aged between 17 and 25 years were randomly assigned to three equal groups	Group 1 (3M Transbond XT): Conventional, three-step adhesive preceded by acid etching Group 2 (3M Transbond Plus): Self-etch primer, mixing acid and primer Group 3 (GC Ortho Connect): One-step adhesive preceded by acid etching	QLF Imaging: at start of treatment (T0), 2 months (T1), and 4 months (T2) Outcomes: ΔF% (mineral loss), WSL surface area (pixels), new lesions, and ΔFMax (%, deepest point) Photographic images taken with Canon EOS 550D: Adjusted image size and orientation Visual and software-based assessment for decalcification signs	Group 2 (3M Transbond Plus) had a significantly higher incidence of WSLs than Group 1 (3M Transbond XT) (p <0.05) Group 3 (GC Ortho Connect) showed a significantly higher incidence than both groups (p < 0.001) Group 3 (GC Ortho Connect) also had larger lesion areas and deeper lesions compared to other groups (p < 0.001)	The lack of primer in the one-step adhesive bonding group contributed to the development of a larger number of and more severe WSLs
Oz et al 2019^24^	Split-mouth, clinical trial	To compare the long-term effectiveness of an antibacterial monomer-containing primer versus a conventional primer for preventing demineralization around brackets. To record the clinical bond failure rates of the brackets	35 patients aged 14.4 years (12 females, 23 males)	Clearfil (CF) group: Used Clearfil Protect Bond with fluoride release and antibacterial properties Transbond (TB) group: Used Transbond XT Primer, a conventional primer. Both groups had patients as their own control using a split-mouth design	WSL severity: Assessment: Digital images were taken before and after orthodontic treatment, and WSL severity was rated from 0 to 3 WSL area calculation: Imaging software calculated the areas of WSLs (Image J version 2.0) Clinical failure rate: Records were kept for first-time bracket failures during treatment	The incidence of WSLs in the Clearfil (CF) group was 8.03%, while in the Transbond (TB) group, it was 9.24% with no significant difference (p = 0.82) No statistically significant difference in bond failure rates was observed between quadrants bonded with Clearfil (CF) and those bonded with the conventional primer (p = 0.316)	Antibacterial monomer primer shows no significant difference in demineralization reduction efficacy
Oz et al 2017^25^	In-vivo study	To compare the effectiveness of an antibacterial fluoride-releasing adhesive, fluoride-recharging adhesive, and conventional orthodontic adhesive in preventing enamel demineralization	15 patients (5 males and 10 females) with an average age of 14.7 years, required orthodontic bicuspid extraction. Total of 45 bicuspids to be examined	The study used three adhesives: Clearfil Protect Bond (fluoride-releasing and antibacterial), Opal Seal (fluoride-releasing), and Transbond XT (conventional). Three premolars per patient were randomly bonded, and after 8 weeks, they were micro-CT scanned post-extraction	Micro-CT scanning: Digital sectional images were acquired and analyzed using ImageJ software	There was no significant difference among the WSL rates of the Clearfil, Opal Seal, and Transbond XT adhesives (p > 0.05) The WSL volume was lower in the Opal Seal group than in the Clearfil and Transbond XT groups, but there was no significant difference among the groups (p > 0.05)	No significant differences found in preventive effects of adhesives over 8 weeks
Tan et al 2020^36^	Split-mouth, clinical trial	To investigate the effects of adhesive precoated (APC) and flash-free (absence of excessive adhesive material) brackets on enamel demineralization	30 patients (20 females and 10 males) between the ages of 12 and 18 years	Using a split-mouth design, both APC flash-free and conventional ceramic brackets were bonded randomly in each patient	DIAGNOdent scores: Categorization based on scores. Measurements were obtained at four sites. Records were obtained immediately after bonding (T0), 1 month after bonding (T1), and 6 months after bonding (T2)	Intergroup evaluation showed that there were no statistically significant differences in demineralization measurements between all sides of the conventional and flash-free brackets at all the time intervals (p >0.05)	APC flash-free and conventional brackets show similar effects on enamel
Yetkiner et al 2019^40^	Randomized controlled clinical trial	To compare adhesive flash-free (FF) and adhesive precoated (APC) brackets in terms of enamel demineralization	50 adolescents (14.23 ± 0.15 years)	Patients randomly distributed to receive (FF) or (APC) ceramic brackets in the maxillary right or left quadrants	QLF imaging: To assess enamel demineralization with a specialized camera Baseline (T0) measurements were taken before bracket placement. Changes in fluorescence (ΔF) were determined by comparing sound and demineralized areas after debonding	ΔF values (T0-debonding) were not statistically significantly different between the groups	Flash-free brackets do not clinically reduce WSL but do reduce pathogenic bacteria

The review included diverse study designs, such as single-center and multicenter randomized clinical trials (RCTs) with variations like double-blind, triple-blinded, split-mouth, and two- or three-arm parallel designs. It also encompassed prospective clinical studies and *in-vivo* investigations. Objectives ranged from comparing bonding systems to assessing the long-term effectiveness of antibacterial primers and the clinical impact of fluoride-releasing adhesives on enamel demineralization during fixed orthodontic treatment.

Our review comprised 550 participants from various studies, ages 12 to 35, representing diverse demographics and involving over 2,000 teeth and tooth surfaces. Interventions featured quadrant-specific primer allocation, diverse adhesive applications, and both direct and indirect bonding methods, showcasing a comprehensive array of orthodontic bonding techniques. Primary evaluation methods included quantitative light-induced fluorescence (QLF) imaging, DIAGNOdent scores, *Streptococcus mutans* counts, photographic images, real-time polymerase chain reaction (PCR), and micro-CT scanning.

### Methodological Approaches of the Studies

Based on methodology, studies can be categorized into three groups. In the first group is where the authors modified the adhesive components, Al Tuma et al incorporated nCaF_2_ into a primer to reduce WSL,^3^ while Alshammari et al added ACP to the adhesive.^6^ Mollabashi et al introduced TiO_2_ nanoparticles to the composite,^23^ and Farzanegan et al combined TiO_2_ and chitosan.^14^ Conversely, other articles explored diverse types of adhesives; Benson et al compared modified glass ionomer to resin adhesive,^8^ Horan et al assessed three bonding systems,^17^ and Oz et al compared antibacterial Clearfil monomer to a conventional adhesive.^24,25^ Comert et al contrasted Opal Seal (fluoride-filled primer) with a conventional primer.^12^ Lastly, studies compared bonding techniques, such as Atilla et al comparing direct and indirect bonding,^7^ Tan et al, and Yetkiner et al evaluating precoated flash-free brackets to conventional techniques.^36,40^

### Main Outcomes and Results

Based on the effectiveness of additives or bonding methods on WSLs during orthodontic treatment, four categories have been identified (Table 1).

#### Effective in reducing WSLs

- *nCaF2-primer:* Effective in suppressing *S. mutans* count and WSLs count with DIAGNOdent.^3^
- *Amorphous calcium phosphate-containing adhesives:* Advantageous in minimizing enamel demineralization.^6^
- Indirect bonding with flowable composites: More effective in inhibiting WSL formation than direct bonding methods.^7^

#### Ineffective in reducing WSLs

Absence of primer in the one-step adhesive bonding group (GC Ortho Connect): Contributes to a higher number and more severe WSLs.^17^

#### Similar effects

Resin-modified glass ionomer: Not more effective than conventional resin bonding in reducing new WSLs.^8^

Fluoride-releasing primers (Opal Seal and Clearfil): Do not demonstrate an advantage in demineralization reduction.^12,24,25^

APC flash-free and conventional brackets: Show similar effects on enamel, with flash-free brackets reducing pathogenic bacteria without a clinical reduction in WSLs.^36,40^

#### Mixed results

TiO2 in two studies shows conflicting results on antibacterial effects.^14,23^

### Risk of Bias in Studies

The ROBINS-I tool was applied for bias assessment in the reviewed studies (Table 2). Comert et al displayed a moderate overall risk, attributed to low bias in most domains but moderate bias due to monthly Opal Seal application in one group.^12^ Oz et al (2019) had a moderate overall risk, with low bias in most domains but introducing a moderate risk of fluor “contamination” in a split-mouth design.^24^ Oz et al (2017) presented a serious overall risk, characterized by a mix of moderate to low bias and serious risk linked to a short intervention period and multiple Opal Seal applications.^25^ Tan et al demonstrated an overall low risk, showcasing low bias across all domains.^36^

**Table 2:** ROBINS-I bias assessment summary

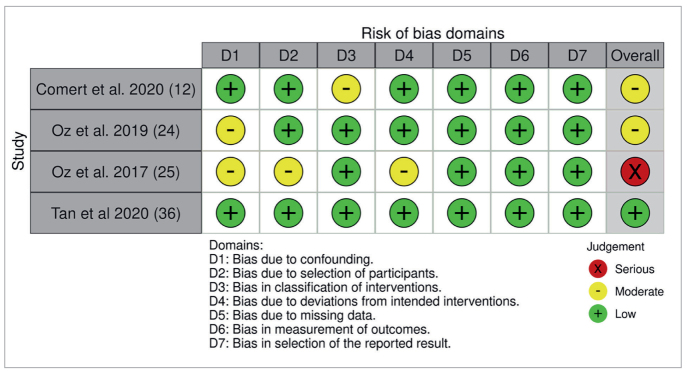

Using the Rob2 tool (Table 3), bias assessment across various studies revealed predominantly low risks, with Alshammari et al,^6^ Farzanegan et al,^14^ Horan et al,^17^ and Yetkiner et al^40^ displaying low risks across all domains, while Atilla et al^7^ and Mollabashi et al^23^ showed some concerns. In contrast, Benson et al^8^ had an overall high risk, primarily associated with confounding and selection bias.

**Table 3:** Rob2 bias assessment overview

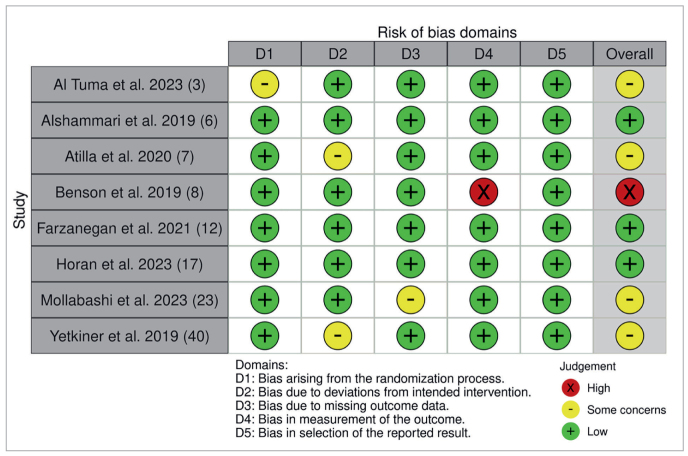

## Discussion

In response to the growing need for patient-independent methods to prevent and address white spot lesions and caries in fixed orthodontic treatments, various studies have explored the modification of orthodontic composites and primers using antimicrobial nanomaterials. While these studies offer insights into potential solutions, it’s crucial to recognize that effective plaque control by the patient remains a cornerstone in preventing WSLs during orthodontic treatment. Plaque accumulation around brackets and wires creates an environment conducive to enamel demineralization, ultimately leading to the formation of WSLs.^21^ Therefore, it’s imperative to emphasize the indispensable role of patient-centered oral hygiene practices to actively lower the risk of WSL development. This underscores the comprehensive approach required to address WSLs in orthodontic care, integrating both patient-centered strategies and innovative material modifications.

### Discussion of the Main Findings

#### Calcium fluoride nanoparticle primer

The included study reveals that the nCaF_2_ primer effectively reduces demineralization and suppresses *S. mutans* following bracket bonding. However, no significant clinical difference was observed at debonding.^12^ While the authors categorized the primer as successful in reducing white spot lesions, caution is warranted due to the split-mouth design. Cross-contamination of fluoride from one side to the other may have occurred, potentially diminishing the clinical significance of WSL prevalence on the control side. This influenced the decision to classify the RoB arising from the randomization process as moderate.

In the context of incorporating nanoparticles into an existing primer, a pertinent question arises about potential effects on cytotoxicity and bond strength. The same authors have addressed this concern in a previous article. In their study, the new primer was found to exhibit acceptable cytotoxicity levels and satisfactory mechanical properties, as demonstrated by testing shear bond strength and an Adhesive Remnant Index.^4^

#### Amorphous calcium phosphate adhesives

The triple-blinded RCT highlights the positive impact of an ACP adhesive on reducing enamel demineralization in early fixed orthodontic treatment, indicating potential benefits for preventing OIWSLs.^6^ Notably, ACP exhibits a beneficial feature: at neutral or high pH levels in the mouth, it remains as ACP. However, during a carious attack when the pH drops to or below 5.8, ACP is converted to hydroxyapatite (HAP) and precipitates, effectively replacing the HAP lost due to acid, which adds a protective element against acidic challenges.^13^ Despite these promising findings, a concern arises from the limited literature on how adding ACP affects the mechanical properties of the adhesive on the enamel bond. Additionally, the study acknowledges limitations, including its short duration relative to the long-term nature of enamel demineralization and incomplete consideration of factors such as oral hygiene, cultural influences, and dietary patterns.

#### Indirect bonding with flowable composites

According to Atilla et al, the indirect bonding (IB) technique, utilizing flowable composite adhesive, appears more effective in preventing white spot lesion formation.^7^ The authors highlight IB’s advantages such as precise bracket placement, reduced chair time, contamination, and treatment duration compared to direct bonding (DB). The authors add that flowable composite, a key component in IB, contributes to its success by facilitating better penetration into acid-etched enamel, resulting in lower Adhesive Remnant Index, and enhanced mechanical adhesion. This composite type also exhibits a reduced risk of bonding failures, decreasing the need for bracket repositioning and rebonding. The study underscores the efficacy of IB in minimizing WSL formation, highlighting the added benefits of flowable composite in achieving superior bonding outcomes during orthodontic treatment. However, these findings are contradicted by a systematic review comparing DB and IB,^20^ indicating insufficient evidence to support the superiority of IB in bracket placement and failure rates, thus challenging the main advantage proposed by Atilla et al.^7^

#### Resin-modified glass ionomer

The study of Benson et al found no evidence that using resin-modified glass-ionomer cement (RM-GIC) over light-cured composite for bonding brackets reduces the incidence of new demineralized lesions or bond failures in fixed orthodontic appliances.^8^ A parallel systematic review and meta-analysis, encompassing five randomized controlled trials and four non-randomized controlled trials, echoed these findings. This comprehensive analysis revealed no significant difference in the incidence of WSLs or bond failure between orthodontic brackets bonded with RM-GIC versus conventional composite.^19^

It appears that, despite promising *in-vitro* tests demonstrating fluoride release, recharge, and subsequent release, there is currently no clinical evidence supporting the use of RM-GIC.

#### Fluoride-releasing primers

Overall, fluoride-releasing primers such as Opal Seal and Clearfil Protect Bond lacked a significant clinical advantage in preventing white spot lesions in the evaluated contexts, suggesting a need for further research to comprehensively assess their efficacy in diverse orthodontic scenarios.^12,24,25^

Future developments in primer technology should focus on optimizing fluoride concentration, release kinetics, and overall efficacy to better address the prevention of WSLs during orthodontic treatment.

#### TiO_2_ nanoparticles

Farzanegan et al chose chitosan and TiO_2_ nanoparticles for orthodontic adhesives due to their antimicrobial properties and biocompatibility. Their study demonstrated a significant reduction in *Streptococcus mutans* counts and enamel mineral content.^14^ In contrast, Mollabashi et al used TiO_2_ for antibacterial and remineralizing effects, showing effective demineralization prevention but not statistically significant antibacterial effects.^23^ Differences in experimental design may explain the contradictions, emphasizing the need for standardized research methods.

Despite both articles asserting the biocompatibility of TiO_2_ nanoparticles, there is other evidence that suggests the contrary. Products for oral use must be biocompatible, raising concerns about the incorporation of titanium particles for enhanced antibacterial effects. A 2016 literature review linked TiO_2_ nanoparticles to oxidative stress, histopathological changes, carcinogenic effects, genotoxicity, and immune system disruptions. Consequently, the use of these materials in human contexts should either be avoided or closely regulated to mitigate potential health risks.^30^

#### Self-adhesive orthodontic resin (GC Ortho Connect™)

Modern adhesive systems, exemplified by those that integrate primer with bonding composite, simplify orthodontic bonding, reducing steps from three to two. This not only saves time, minimizes errors, and decreases residual adhesive on enamel post-debonding,^11^ potentially averting white spot lesion development – an objective in Horan et al’s randomized controlled trial.^17^ However, the study implies that the absence of primer contributed to more numerous and severe WSLs. Thus, the findings suggest that lacking primer may not prevent WSL occurrence and may even lead to more significant lesions during fixed orthodontic therapy. Particularly, the one-step adhesive system group exhibited the highest number of newly developed WSLs, mineral loss, and lesion area, highlighting the negative impact of the absence of the primer layer on etched enamel.

#### Adhesive precoated flash-free brackets

The study of APC flash-free brackets is of interest due to recent advancements in material science and the introduction of adhesive precoated brackets that eliminate the need for excessive adhesive removal.^15^ These brackets use a nonwoven mat soaked with low-viscosity adhesive resin, providing benefits such as shorter chair time, adequate bond strength, and quicker clean-up. Additionally, researchers have explored potential advantages, including protective effects against demineralization, and improved oral hygiene by reducing retentive sites for plaque accumulation.^16^ Clinical trials by Tan et al revealed no significant differences in enamel demineralization and periodontal health between APC flash-free and conventional brackets.^36^ The study by Yetkiner et al found comparable plaque quantity but a composition with fewer pathogenic bacteria around adhesive flash-free brackets compared to conventional brackets.^40^

Despite the APC flash-free brackets having several advantages, to this date, research has not established any added value in using APC flash-free brackets for high-risk patients prone to white spot lesions.

### Evaluation Methods

Various methods are used to assess demineralization lesions during fixed orthodontic treatment, each with its own advantages and disadvantages.

Clinical examinations, which directly observe and evaluate the teeth, provide a practical and real-time assessment. However, maintaining consistency among different examiners throughout a lengthy clinical trial can be challenging, impacting reliability.^27^

Photographic evaluations offer the advantage of allowing clinically relevant assessments of before-and-after images, enabling multiple masked assessors to evaluate simultaneously. This method avoids the need for continuous calibration of clinical judges but relies on subjective visual judgment. The use of multiple assessors is essential to enhance reliability, as unanimous agreement may be challenging to achieve.^8^

Fluorescent techniques, such as QLF, provide a sensitive and quantitative approach, validated against destructive methods like transverse microradiography. QLF can detect demineralization early, even before it is visually apparent. However, it may be overly sensitive, potentially overestimating the prevalence of demineralization. Additionally, specialized equipment is required.^2^

Microcomputed tomography (micro-CT), a three-dimensional analysis method, calculates lesion volumes without damaging sample surfaces, enabling repeated scanning and sensitivity measurements. However, its drawbacks, including the necessity for tooth extraction and limitations in non-extraction orthodontic treatment, along with high costs, long scanning times, and extended data analysis, limit its generalizability.^25^

Real-time PCR is a valuable method for counting *S. mutans* and offers distinct advantages in microbial analysis. Its quantitative nature enables the precise measurement of bacterial abundance. However, drawbacks of real-time PCR include cost, susceptibility to inhibitors, specialized equipment needs, potential contamination risk, and the complexity of data analysis, limiting its widespread use. The limited correlation with clinical significance and changes in white spot lesions is a major limitation. The technique primarily focuses on quantitative measures, often lacking a direct reflection of the actual impact on oral health outcomes and treatment efficacy.^23^

Due to methodological differences and varying sensitivities, a bonding method may appear more or less effective in preventing white spot lesions. Attention to proposed results is crucial for accurate interpretation.

### Limitations of Evidence

#### Assessing methodological heterogeneity and intervention variability

The RoB assessments employing ROBINS-I and Rob2 tools suggest heterogeneous methodological quality among studies. Those with low risk, exemplified by Tan et al,^36^ likely yield more dependable results. Conversely, studies with moderate or serious risk, like Oz et al,^25^ may possess limitations affecting result reliability. High-risk studies, including Benson et al,^8^ raise substantial concerns about internal validity and result credibility, underscoring the importance of discernment when interpreting findings across the spectrum of bias in these intervention studies.

Moreover, upon thorough review, a meta-analysis was not performed due to pronounced heterogeneity in intervention across the included studies. The diverse nature of interventions, ranging from fluoride addition to various adhesive agents, introduces a substantial level of variability. Additionally, the use of different evaluation methods across studies further contributes to methodological diversity, making it challenging to draw meaningful comparisons and synthesize results in a comprehensive manner.

### Evaluation of the Review Process

The methodology demonstrates robustness by adhering to PRISMA guidelines, incorporating a comprehensive protocol, and ensuring transparency through PROSPERO registration. Well-defined inclusion/exclusion criteria, diverse information sources, and a comprehensive search strategy enhance precision. The dual-reviewer screening process, consistent data extraction, and clear outcome delineation add rigor. Specific RoB assessment tools tailored to study types reflect a nuanced approach. These methodological strengths collectively contribute to the reliability, transparency, and comprehensiveness of our systematic review.

### Other Emerging Nanoparticles

In addition to the reviewed literature, various nanoparticles are undergoing *in-vitro* testing for their efficacy in preventing white spot lesions and inhibiting bacterial growth in orthodontic applications. Zinc oxide nanoparticles (ZnO NPs) and Chitosan Nanoparticles have demonstrated notable antibacterial effects against *S. mutans*, *S. sanguis*, and *Lactobacillus acidophilus.*^42^ Copper oxide nanoparticles (CuO NPs) exhibit antimicrobial properties in orthodontic adhesives.^37^ Furthermore, ongoing studies explore the potential of nanoparticles like silver/hydroxyapatite (Ag/HA NPs)^31^ and curcumin nanoparticles (curcumin NPs) for their antimicrobial activities,^32^ indicating a promising field to prevent WSLs in orthodontic patients. Continued research is crucial for advancing these potential applications.

Alongside the examined nanoparticles, ongoing *in-vitro* investigations assess the effectiveness of orthodontic bonding resins enriched with bioactive glass for remineralizing enamel and managing white spot lesions during orthodontic treatment. A systematic review studied the efficacy of such resins, encompassing seven *in-vitro* studies. This review consistently showcased the superiority of bioactive glass resins in promoting remineralization compared to the control group. While producing positive results, the review noted a limitation, primarily involving *in-vitro studies*, emphasizing the necessity for standardized *in-vivo* studies with a more uniform protocol to reduce heterogeneity.^5^

### Considerations Beyond WSLs Prevention

To our knowledge, this literature review is the first to underscore the importance of adhesive selection based on patient-specific risk factors, aiming to find a balance between demineralization control and orthodontic treatment success. While the effectiveness of orthodontic adhesives and primers in preventing WSLs is a crucial factor, it should not be the only consideration in choosing bonding materials. Inadequate bond strength may result in frequent bracket-to-enamel bond failures, while excessive bond strength can potentially harm the enamel surface during debonding.^10^ Reynolds recommends a minimum bond strength ranging from 5.9 to 7.9 MPa for successful clinical bonding.^29^ Unfortunately, the shear bond strength is not sufficiently emphasized in the included articles. This is a very important criterion because even if an adhesive proves to be more effective in reducing WSLs, its utility diminishes if the addition of antibacterial agents negatively influences bonding performance.

Moreover, products intended for use in the oral cavity should be biocompatible and devoid of potential harm. This is particularly noteworthy when incorporating nanoparticles aimed at enhancing antibacterial effects, which may not always align with the criteria of non-toxicity and biocompatibility.

## Conclusion

In conclusion, various nanoparticles, such as nCaF_2_, ACP, and TiO_2_ may play a role in preventing white spot lesions during fixed orthodontic treatment. Evaluation methods, including clinical examinations, photographic assessments, and advanced imaging techniques, impact result interpretation. The effectiveness of orthodontic adhesives in WSL prevention should consider patient-specific factors, balancing demineralization control and bond strength. While ongoing research explores additional nanoparticles, caution is needed to ensure biocompatibility and adherence to safety standards in oral applications.

## References

[ref1] Aimutis WR (2004). Bioactive properties of milk proteins with particular focus on anticariogenesis. J Nutr.

[ref2] Al Maaitah EF, Adeyemi AA, Higham SM, Pender N, Harrison JE (2011). Factors affecting demineralization during orthodontic treatment: a post-hoc analysis of RCT recruits. Am J Orthod Dentofacial Orthop.

[ref3] Al Tuma RR, Yassir YA (2023). Effect of calcium fluoride nanoparticles in prevention of demineralization during orthodontic fixed appliance treatment: a randomized clinical trial. Eur J Orthod.

[ref4] Al Tuma RR, Yassir YA (2021). Evaluation of a newly developed calcium fluoride nanoparticles-containing orthodontic primer: an in-vitro study. J Mech Behav Biomed Mater.

[ref5] Alamri A, Salloot Z, Alshaia A, Ibrahim MS (2020). The effect of bioactive glass-enhanced orthodontic bonding resins on prevention of demineralization: a systematic review. Molecules.

[ref6] Alshammari FM, Sanea JA (2019). Efficacy of amorphous calcium phosphate (ACP) containing adhesive in preventing demineralization during orthodontic treatment, a triple blinded randomized clinical trial (RCT). J Contemp Dent Pract.

[ref7] Atilla AO, Ozturk T, Eruz MM, Yagci A (2020). A comparative assessment of orthodontic treatment outcomes using the quantitative light-induced fluorescence (QLF) method between direct bonding and indirect bonding techniques in adolescents: a single-centre, single-blind randomized controlled trial. Eur J Orthod.

[ref8] Benson PE, Alexander-Abt J, Cotter S, Dyer FMV, Fenesha F, Patel A (2019). Resin-modified glass ionomer cement vs composite for orthodontic bonding: a multicenter, single-blind, randomized controlled trial. Am J Orthod Dentofacial Orthop.

[ref9] Benson PE, Parkin N, Dyer F, Millett DT, Furness S, Germain P (2013). Fluorides for the prevention of early tooth decay (demineralised white lesions) during fixed brace treatment. Cochrane Database Syst Rev.

[ref10] Bilal R, Arjumand B (2019). Shear bond strength and bonding properties of orthodontic and nano adhesives: a comparative in-vitro study. Contemp Clin Dent.

[ref11] Choi A, Yoo K-H, Yoon S-Y, Park S-B, Choi Y-K, Kim Y-I (2022). Enhanced antimicrobial and remineralizing properties of self-adhesive orthodontic resin containing mesoporous bioactive glass and zwitterionic material. J Dent Sci.

[ref12] Comert S, Oz AA (2020). Clinical effect of a fluoride-releasing and rechargeable primer in reducing white spot lesions during orthodontic treatment. Am J Orthod Dentofacial Orthop.

[ref13] Cross KJ, Huq NL, Stanton DP, Sum M, Reynolds EC (2004). NMR studies of a novel calcium, phosphate and fluoride delivery vehicle-αS1-casein(59–79) by stabilized amorphous calcium fluoride phosphate nanocomplexes. Biomaterials.

[ref14] Farzanegan F, Shahabi M, Niazi AE, Soleimanpour S, Shafaee H, Rangrazi A (2021). Effect of the addition of chitosan and TiO_2_ nanoparticles on antibacterial properties of an orthodontic composite in fixed orthodontic treatment: a randomized clinical trial study. Biomed Phys Eng Express.

[ref15] Foersch M, Schuster C, Rahimi RK, Wehrbein H, Jacobs C (2016). A new flash-free orthodontic adhesive system: a first clinical and stereomicroscopic study. Angle Orthod.

[ref16] Grünheid T, Larson BE (2018). Comparative assessment of bonding time and 1-year bracket survival using flash-free and conventional adhesives for orthodontic bracket bonding: a split-mouth randomized controlled clinical trial. Am J Orthod Dentofacial Orthop.

[ref17] Horan OGA, Al-Khateeb SN (2023). Comparison of three orthodontic bonding systems in white spot lesion development: a randomized clinical trial. Angle Orthod.

[ref18] Khachatryan G, Markaryan M, Vardanyan I, Manrikyan M, Manrikyan G (2022). Morphological characteristics and prevention of tooth enamel demineralization during orthodontic treatment with non-removable appliances. Int J Environ Res Public Health.

[ref19] Khan AR, Fida M, Gul M (2020). Decalcification and bond failure rate in resin modified glass ionomer cement versus conventional composite for orthodontic bonding: a systematic review & meta-analysis. Int Orthod.

[ref20] Li Y, Li Mei, Wei J, Yan X, Zhang X, Zheng W, Li Y (2019). Effectiveness, efficiency and adverse effects of using direct or indirect bonding technique in orthodontic patients: a systematic review and meta-analysis. BMC Oral Health.

[ref21] Lopatiene K, Borisovaite M, Lapenaite E (2016). Prevention and treatment of white spot lesions during and after treatment with fixed orthodontic appliances: a systematic literature review. J Oral Maxillofac Res.

[ref22] Marinelli G (2021). White spot lesions in orthodontics: prevention and treatment. A descriptive review. J Biol Regul Homeost Agents.

[ref23] Mollabashi V, Soleymani M, Arabestani MR, Farhadian M, Abbasalipourkabir R, Salehzadeh M (2023). Evaluation of nano TiO_2_ modified orthodontic composite effects on *S. mutans* population and enamel demineralization in fixed orthodontic patients; a split mouth randomized controlled clinical trial. Biol Trace Elem Res.

[ref24] Oz AZ, Oz AA, Yazicioglu S, Sancaktar O (2019). Effectiveness of an antibacterial primer used with adhesive-coated brackets on enamel demineralization around brackets: an *in vivo* study. Prog Orthod.

[ref25] Oz AZ, Oz AA, Yazıcıoglu S (2017). *In vivo* effect of antibacterial and fluoride-releasing adhesives on enamel demineralization around brackets: a micro-CT study. Angle Orthod.

[ref26] Page MJ, McKenzie JE, Bossuyt PM, Boutron I, Hoffmann TC, Mulrow CD (2021). The PRISMA 2020 statement: an updated guideline for reporting systematic reviews. BMJ.

[ref27] Pretty IA, Ekstrand KR (2016). Detection and monitoring of early caries lesions: a review. Eur Arch Paediatr Dent.

[ref28] Puleio F, Fiorillo L, Gorassini F, Iandolo A, Meto A, D’Amico C (2022). Systematic review on white spot lesions treatments. Eur J Dent.

[ref29] Reynolds IR, Von Fraunhofer JA (1976). Direct bonding of orthodontic brackets – a comparative study of adhesives. Br J Orthod.

[ref30] Shakeel M, Jabeen F, Shabbir S, Asghar MS, Khan MS, Chaudhry AS (2016). Toxicity of nano-titanium dioxide (TiO2-NP) through various routes of exposure: a review. Biol Trace Elem Res.

[ref31] Sodagar A, Akhavan A, Hashemi E, Arab S, Pourhajibagher M, Sodagar K (2016). Evaluation of the antibacterial activity of a conventional orthodontic composite containing silver/hydroxyapatite nanoparticles. Prog Orthod.

[ref32] Sodagar A, Bahador A, Pourhajibagher M, Ahmadi B, Baghaeian P (2016). Effect of addition of curcumin nanoparticles on antimicrobial property and shear bond strength of orthodontic composite to bovine enamel. J Dent Tehran Iran.

[ref33] Srivastava K, Tikku T, Khanna R, Sachan K (2013). Risk factors and management of white spot lesions in orthodontics. J Orthod Sci.

[ref34] Sterne JA, Hernán MA, Reeves BC, Savović J, Berkman ND, Viswanathan M (2016). ROBINS-I: a tool for assessing risk of bias in non-randomised studies of interventions. BMJ.

[ref35] Sterne JAC, Savović J, Page MJ, Elbers RG, Blencowe NS, Boutron I (2019). RoB 2: a revised tool for assessing risk of bias in randomised trials. BMJ.

[ref36] Tan A, Çokakoğlu S (2020). Effects of adhesive flash-free brackets on enamel demineralization and periodontal status. Angle Orthod.

[ref37] Toodehzaeim MH, Zandi H, Meshkani H, Hosseinzadeh Firouzabadi A (2018). The effect of CuO nanoparticles on antimicrobial effects and shear bond strength of orthodontic adhesives. J Dent Shiraz Iran.

[ref38] Tufekci E, Dixon JS, Gunsolley JC, Lindauer SJ (2011). Prevalence of white spot lesions during orthodontic treatment with fixed appliances. Angle Orthod.

[ref39] Tüfekçi E, Pennella DR, Mitchell JC, Best AM, Lindauer SJ (2014). Efficacy of a fluoride-releasing orthodontic primer in reducing demineralization around brackets: an in-vivo study. Am J Orthod Dentofacial Orthop.

[ref40] Yetkiner E, Gürlek Ö, Işık A, Lappin DF, Buduneli N (2019). Do adhesive flash-free brackets affect bacterial plaque in patients with adequate oral hygiene? A randomised controlled clinical and microbiological assessment. Oral Health Prev Dent.

[ref41] Yetkiner E, Gürlek Ö, Işık A, Lappin DF, Buduneli N (2019). Do adhesive flash-free brackets affect bacterial plaque in patients with adequate oral hygiene? A randomised controlled clinical and microbiological assessment. Oral Health Prev Dent.

[ref42] Mirhashemi A, Bahador A, Kassaee M, Daryakenari G, Ahmad-Akhoundi M, Sodagar A (2013). Antimicrobial effect of nano-zinc oxide and nano-chitosan particles in dental composite used in orthodontics. J Med Bacteriol.

